# A numerical investigation to design and performance optimization of lead-free Cs_2_TiCl_6 _based perovskite solar cells with different charge transport layers

**DOI:** 10.1038/s41598-025-06820-1

**Published:** 2025-07-01

**Authors:** Sahjahan Islam, M. Khalid Hossain, M. Shihab Uddin, P. Prabhu, Suhas Ballal, K. Phaninder Vinay, V. Kavitha, Satish Kumar Samal, Abdullah M. S. Alhuthali, Mongi Amami, A. Kumar Datta, Gazi F. I. Toki, Rajesh Haldhar

**Affiliations:** 1 Department of Physics & Astronomy, East Texas A&M University-Commerce, Commerce, TX 75428 USA; 2 Institute of Electronics, Atomic Energy Research Establishment, Bangladesh Atomic Energy Commission, Dhaka, 1349 Bangladesh; 3 Department of Advanced Energy Engineering Science, Interdisciplinary Graduate School of Engineering Sciences, Kyushu University, Fukuoka, 816- 8580 Japan; 4 Department of Computer Science and Engineering, Daffodil International University, Dhaka, 1216 Bangladesh; 5 Research and Innovation Cell, Rayat Bahra University, Mohali, Punjab 140301 India; 6 Department of Mechanical Engineering, Mattu University, Mettu, 318, Ethiopia; 7 Department of Chemistry and Biochemistry, School of Sciences, JAIN (Deemed to be University), Bangalore, Karnataka India; 8 Department of ECE, Raghu Engineering College, Visakhapatnam, Andhra Pradesh 531162 India; 9 Department of Chemistry, Sathyabama Institute of Science and Technology, Chennai, Tamil Nadu India; 10 Department of Electronics & Communication Engineering, Siksha ’O’ Anusandhan (Deemed to be University), Bhubaneswar, Odisha 751030 India; 11 Department of Physics, College of Sciences, Taif University, P.O. Box 11099, Taif, 21944 Saudi Arabia; 12 Department of Chemistry, College of Science, King Khalid University, P.O. Box 9004, Abha, 62217 Saudi Arabia; 13 Department of Electrical and Electronic Engineering, Mymensingh Engineering College, Mymensingh, 2200 Bangladesh; 14 Nanotechnology Center, School of Fashion and Textiles, The Hong Kong Polytechnic University, Kowloon, 999077 Hong Kong; 15 School of Chemical Engineering, Yeungnam University, Gyeongsan, 38541 Republic of Korea

**Keywords:** Perovskite solar cell, Cs_2_TiCl_6_ absorber, CdS ETL, CdTe HTL, SCAPS-1D, Engineering, Materials science, Physics

## Abstract

Among the most attractive light-absorbing materials, halide perovskites have been gaining popularity for their versatile range of use in solar cells, lasers, and photodetectors. Whereas, Titanium (Ti)-based all-inorganic perovskite solar cells (PSCs) have garnered attention for their optoelectronic capabilities in response to this situation. In this theoretical study, Cesium Titanium (IV) Halide based lead-free, eco-friendly, and stable Cs_2_TiCl_6_-based PSC has been proposed and a numerical simulation using SCAPS-1D has been carried out to enhance the cell performance by optimizing the device parameters. A different set of hole transport layers (HTLs) like MoO_3,_ ZnTe, CNTS, CuAlO_2,_ CdTe, nPB, C_6_TBTAPH_2,_ N: TiO_2,_ NiCo_2_O_4,_ and PBTTT-C14 was simulated in combination with electron transport layers(ETLs) such as CdS, Nb_2_O_5_, ZnSe, and MZO. After several cell optimizations like thickness, acceptor, donor, and defect concentration of selected four structures, the best cell structure are suggested e.g., FTO/CdS/Cs_2_TiCl_6_/CdTe/Au that shows a PCE of 18.15% along with the short circuit current density (*J*_SC_) of 17.83 mA/cm^2^, open-circuit voltage (*V*_OC_) of 1.188 V, fill factor (FF) of 89.51%. Among all devices, the solar cell performance decreases when series resistance (*R*_S_) and temperature are increased as opposed to shunt resistance (*R*_Sh_). The obtained results reveal that Cs_2_TiCl_6_-based PSC can contribute to the advancement of efficient non-toxic, all-organic perovskite solar cells in the future.

##  Introduction

A significant proportion of residential areas in the contemporary global context get uninterrupted exposure to sunlight throughout the year. Energy generated from the sun is abundant, and it provides efficient energy that makes solar power energy a good alternative to limited fossil fuels to mitigate the future energy demand^1^. Scientists are conducting various kinds of research to discover the most effective, environment-friendly, low-cost, etc. devices for renewable energy resources^2^. Among them, photovoltaic (PV) cells are considered top-notch among these sustainable energy sources, possessing the complete capability to fulfill the aforementioned criteria^3–5^.

As regards the production of solar energy, silicon-based solar cells (SCs) have cemented their top position with a share of 95% in the present PV market with laboratory power-conversion efficiency (PCE) over 25%^6^. Despite these extensive advancements in SC technology, the production costs related to Si-based solar cells could not be minimized due to the usage of cleanroom technology, thus increasing the costs of those applications^6,7^. When researchers worldwide are in search of an alternative to silicon solar cells, halide-based perovskite solar cells (PSCs) have appeared as a replacement, showing exceptional photoelectric implementation, higher electrical constraints for instance quantum efficiency (QE), current density, and lower manufacturing rate^8–11^. Recent studies have shown an increment of using lead (Pb) halide PSCs, recorded PCE of 25.6%^12^, because of their ideal bandgap, affordability, higher absorption coefficient, and long carrier diffusion length. However, there are two main concerns for the commercial applications of Pb-based PSCs e.g. (i) the toxicity of lead affects the ecosystem, and (ii) the instability of devices caused by organic citations^13,14^. In these circumstances, Tin (Sn^+2^) ^15,16^, Germanium (Ge^+2^)^17^, Bismuth (Bi^+3^) ^18,19^, and Palladium (Pd^+4^)^20^ are considered replacement of toxic lead in practical SC usage. However, it was reported by Babayigit et al. that Sn-based halide perovskites also are responsible for large amounts of poisonous elements^21^. Moreover, upon contact with air, Sn^+2^ also transforms to Sn^+4^ for oxidation. Thus, a special kind of PSCs has been a subject of interest for researchers in finding stable and non-toxic perovskites^22^.

A novel group of halide perovskites, centered around Titanium (Ti) (IV), and particularly exemplified by Cs_2_TiCl_6_, has been documented as a potential material for the field of SC applications where Ti is a substitution of hazardous Pb. Because of the enduring oxidation state stability of titanium, Cs_2_TiCl_6_ can absorb thermal stress and can be utilized in challenging hazy environments making it the best candidate for Pb-free PSCs. Chakraborty et al. examined the Cs_2_TiCl_6_ absorbing layer and revealed its thickness and indirect band gap are 1.0 μm and 2.9 eV, respectively^23^. The report analyzed the electronic and optical properties of Ti-based PSC and illustrated that Cs_2_TiCl_6_ is compatible with PV and optoelectronic implementations^24^. While Moiz et al. optimized his proposed device and showed a maximum PCE of roughly over 18.5%^4^. A contrastive analysis was conducted by Mokhtari et al. and found that Cs_2_TiCl_6_ indicates a high absorption coefficient of 10^5^ cm^−1^ in the visible light region ^25^. There have been few investigations conducted on Cs_2_TiCl_6_, and determining its optimal device architectures has been an appealing subject for experts in recent years. In order to achieve an effective PV response, the perovskite absorber layer positioned itself between the charge transport layers like the electron transport layer (ETL) and hole transport layer (HTL). Most importantly, the highest PV can be attained when the charge carriers are distributed uniformly within SCs^7,26^. The outcome of PSCs is extensively determined by the ETL and HTL since these layers play a significant role in various processes such as charge carrier extraction, transport, and recombination. Thus, It is of paramount importance to select the ETL with suitable band alignment, excellent electron mobility, sufficient light transmittance, and resistance to moisture^27–29^. In this work, four ETLs such as cadmium sulfide (CdS), Niobium pentoxide (Nb_2_O_5_), Zinc selenide (ZnSe), Magnesium zinc oxide (MZO) in conjunction with preferred and compatible HTLs like as Nitrogen-Doped Titanium Dioxide (N: TiO_2_)^30^, n-Propyl Bromide (nPB)^31^, Nickel cobaltite (NiCo_2_O_4_), Molybdenum trioxide (MoO_3_)^32^, Poly(2,5-bis(3-tetradecylthiophen-2yl)thieno(3,2-b)thiophene) (PBTTT-C14)^33^, Copper Aluminum Oxide (CuAlO_2_)^34^, Zinc telluride (ZnTe)^35^, Carbon nanotubes (CNTS)^36^, Copper zinc tin sulfide (CZTS), Cadmium telluride (CdTe)^37^, Octahexyltetrabenzo Triazaporphyrin (C_6_TBTAPH_2_)^38^ are optimized to figure out the best combinations of PSC. The selection of ETL and HTL materials in this study was guided by several key factors, including band alignment with Cs₂TiCl₆, chemical stability, and interface compatibility. CdS^39^, Nb_2_O_5_^40^_,_ ZnSe^41^, and MZO^42^ were chosen as potential ETLs due to their conduction band levels being well-aligned with the conduction band minimum of Cs_2_TiCl_6_, promoting efficient electron extraction. In addition, these materials are known for their thermal and environmental stability, and have been successfully employed in similar n–i–p architectures. CdTe^43^ was selected as the HTL because of its deep valence band, which supports effective hole transfer from Cs_2_TiCl_6_, while blocking electrons, thereby enhancing charge selectivity and minimizing recombination losses.

The experimental feasibility of Cs_2_TiX_6_-based perovskites has been increasingly validated by recent studies. Chakraborty et al. successfully synthesized Cs_2_TiX_6_ (X = Cl, Br, I, F) compounds and demonstrated through comparative experimental and DFT investigations that Cs_2_TiBr_6_ and Cs_2_TiCl_6_ exhibit favorable bandgaps, material stability, and visible light absorption, with TEM and SAED analyses confirming high crystallinity^44^. Furthermore, Chen et al. reported the fabrication of high-quality Cs_2_TiBr_6_ thin films via a low-temperature vapor-based method, achieving excellent intrinsic and environmental stability, long carrier diffusion lengths, and stable photovoltaic performance with efficiencies up to 3.3%^45^. Additionally, Ju et al. demonstrated that Ti-based vacancy-ordered double perovskite halides such as Cs_2_TiI_x_Br_6−x_ possess tunable bandgaps (1.38–1.78 eV), high stability, benign defect properties, and strong optical absorption, making them highly promising for single-junction and tandem solar cell applications^46^. These experimental advances highlight the viability of synthesizing Cs_2_TiCl_6_-based devices and provide a strong foundation for their further development in eco-friendly, stable, lead-free photovoltaic technologies.

With the desire to build environment-friendly, non-poisonous, comparatively stable, highly efficient SCs, the one-dimensional solar cell capacitance simulator software, SCAPS 1D^47–50^, is employed to lead a thorough assessment of different parameters of PV performance for Cs_2_TiCl_6_-based PSC. This work initially examined 10 compatible HTLs that were optimized to determine the most favorable results, while keeping the CdS ETL constant. The resulting structure was then compared with additional ETLs, namely Nb_2_O_5_, ZnSe, and MZO. Subsequently, the thickness of the absorber and HTL, acceptor density and defect density of the absorber, donor density of ETL, and acceptor density of the HTL are tuned in a sequential manner. Simultaneously, the impact of absorber and HTL thickness, as well as absorber defect and acceptor density, on the PV characteristics is examined. Moreover, the enhanced performance of the four devices is supported by evaluating their current-voltage density, quantum efficiency, resistance, operating temperature, and generation and recombination rate. The device structure that produced the most outstanding PV parameters is refined further to surpass the efficiency of the PSC configuration.

##  Numerical simulations

It was discussed above that the SCAPS-1D is a one-dimension (1-D) PV simulator advanced with C programming language was designed by Marc Bargeman and his co-workers at the University of Gent, Ghent, Belgium)^51–53^. A seven-layer SC stack has been developed into this programming software thus including some significant PV parameters (like thickness, defects, doping density, etc.)^51,54^. The SCAPS-1D has been employed for simulating the PSCs in previous literature^55–59^. The mechanism of this software is fundamentally established on four sets of PV equations, i.e., Poisson equation, continuity equations, charge transport equations, and absorption coefficient equation (for both electrons and holes) are described as follows:

The Poisson equation (Eq. 1) implies the behavior of electrical potential (φ) that includes how different types of electrical charges are circulated inside of SC. The electric charge constant is defined as *q*, it is valued at 1.602 × 10^−19^ C. While the absolute dielectric constant, relative dielectric constant of each layer’s material, donor/acceptor doping density, hole/electron density distribution, and the hole/electron density distribution (in terms of thickness x) are classified with $\:{\epsilon\:}_{0}$, $\:{\epsilon\:}_{r}$, $\:{N}_{\text{A}}/{N}_{D}$, $\:{\rho\:}_{p}/{\rho\:}_{n}$ and p(x)/n(x), respectively.

1 $$\:v\frac{{d}^{2}\:{\upvarphi\:}\left(\text{x}\right)}{d{x}^{2}}=\left(\frac{q}{{\epsilon\:}_{0}{\epsilon\:}_{r}}\right)(\:p\left(x\right)-n\left(x\right)+{N}_{D}-{N}_{A}+\:{\rho\:}_{p}-{\rho\:}_{n})$$.

The derivative of hole current density ($\:{J}_{\text{P}}$) and electron current density ($\:{J}_{\text{n}}$) with respect to position variable x is denoted in terms of carrier generation (*G*), and carrier recombination (*R*) known as the continuity equation (Eqs. 2 and 3).

2 $$\:\frac{d{\text{J}}_{\text{n}}}{dx}=G-R$$

3 $$\:\frac{d{\text{J}}_{\text{P}}}{dx}=G-R$$.

The charge transport equations can be written as follows Eqs. 4 and 5 where µ_p_ (hole) and µ_n_ (electron) are free charge carrier mobility. Meanwhile, electron and hole charge diffusion co-efficient are distinguished with $\:{D}_{\text{n}}$ and $\:{D}_{\text{P}}$ in the order given in Eqs. 4 and 5.

4 $$\:{\text{J}}_{\text{n}}={D}_{\text{n}}\frac{\text{d}\text{n}}{\text{d}\text{x}}+{{\upmu\:}}_{\text{n}}\:\frac{\text{d}\varnothing\:}{\text{d}\text{x}}$$

5 $$\:{\text{J}}_{\text{P}}={D}_{\text{P}}\frac{\text{d}\text{p}}{\text{d}\text{x}}+{{\upmu\:}}_{\text{P}}\:\frac{\text{d}\varnothing\:}{\text{d}\text{x}}$$.

With regards to projecting the absorption coefficient, several designs are available in the algorithm of SCAPS-1D software because of the presence of varieties of semiconducting materials. The optical absorption coefficient model for PSCs can be graded in mathematical ways like Eq. 6, where A, and B are constant, h = 6.62607015 × 10^−34^ JHz^−1^, ν is the frequency of photons and $\:{E}_{\text{g}}$ is the absorber layer’s band gap.:

6 $$\alpha (\lambda )~ = ~(A~ + ~~\frac{B}{{h\vartheta }}\sqrt {h\vartheta - Eg} ~)$$.

Therefore, utilizing the optimum conditions and the above equations SCAPS-1D is used in this simulation to determine the parameters of suggested PSCs such as open-circuit voltage (*V*_OC_), short-circuit current density (*J*_SC_), fill factor (FF), PCE, and QE.

###  Cs_2_TiCl_6_-based PSC structure

**Fig. 1:**
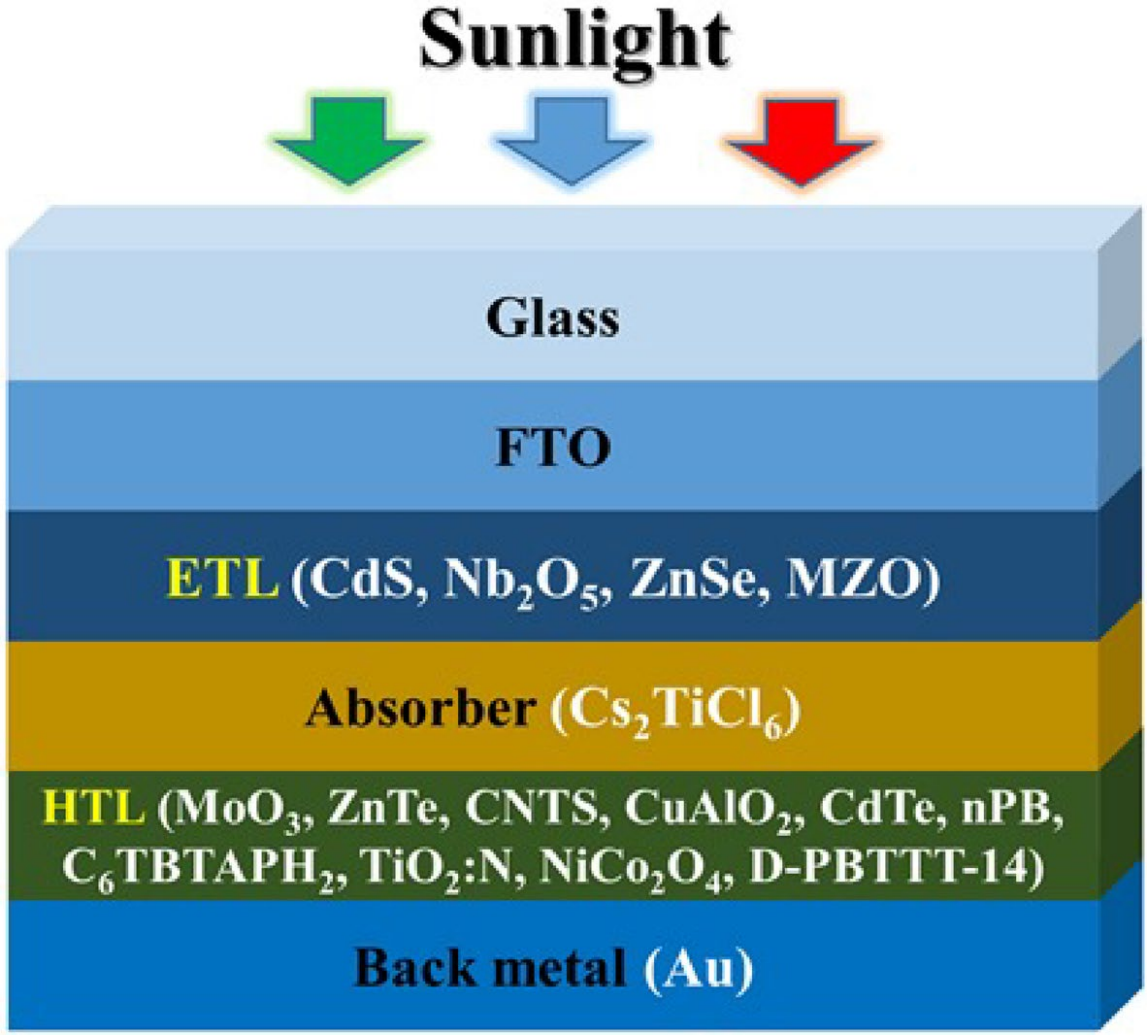
Device Configuration of Cs_2_TiCl_6_-based multilayered PSCs.

The architecture of the layered Cs₂TiCl₆-based solar cells investigated in this study follows an n–i–p planar heterojunction configuration, as illustrated in Fig. 1. In this design, ETL is positioned in the n-region, the perovskite absorber layer (Cs_2_TiCl_6_) occupies the intrinsic (i) region, and HTL is located in the p-region. Upon solar illumination, the perovskite layer generates excitons—bound electron-hole pairs. The dissociation of these excitons occurs primarily at the interfaces between the i-region and the adjacent n- and p-regions, where built-in electric fields assist in separating the charge carriers. Specifically, electrons are driven toward the ETL (n-region), while holes migrate toward the HTL (p-region). The effective separation and transport of these carriers are governed by their respective diffusion lengths and the internal electric field, ensuring efficient charge collection at the corresponding electrodes. The charge transfer characteristics at the HTL/perovskite interface critically influence the efficiency and stability of perovskite solar cells (PSCs). Interfaces can be smooth or rough, with roughness potentially causing charge trapping, increased recombination, and reduced carrier mobility. While smooth interfaces generally promote efficient carrier transport, moderate roughness does not always degrade performance. For instance, Richter et al. showed that moderate interface roughness in CIGSSe solar cells improved optical absorption and carrier collection, leading to enhanced Jsc, Voc, and FF despite slightly higher recombination. Rough interfaces also increase junction capacitance, which must be considered in device analysis^8^. Other studies have intentionally introduced controlled roughness to enhance light scattering and absorption, though excessive roughness can negatively impact charge transport^60^.

Titanium-based Pb-free halide PSCs, Cs_2_TiCl_6_, gold (Au), and Fluorine-doped Tin Oxide (FTO) are employed as an absorber, back contact metal, and transparent conductive oxide (TCO), individually during that experimentation. Furthermore, to determine the best structure for the PSC, four ETLs (such as CdS, Nb_2_O_5_, ZnSe, and MZO) and 10 HTLs (like MoO_3,_ ZnTe, CNTS, CuAlO_2,_ CdTe, nPB, C_6_TBTAPH_2,_ N-TiO_2,_ NiCo_2_O_4,_ PBTTT-C14) are investigated from 36 various combinations to determine the best structure for the PSC. A stable version 3.3.10 of SCAPS-1D is implemented for device modeling and optimization of PSC under room temperature of 300 K and AM.1.5 solar spectrum (light intensity 1000 W/m^2^) in this study. The input parameters used in this simulation are given in Tables 1, 2 and 3. The material parameters used in this simulation, including bandgap energy, electron affinity, dielectric constant, carrier mobilities, and defect densities, were primarily adopted from experimentally reported studies and validated computational research^61,62^. This approach ensures that the numerical results accurately represent realistic device behavior and maintain consistency with observed material properties.

**Table 1:** Properties of FTO, perovskite (Cs_2_TiCl_6_) and etls.

Material property	FTO	Cs_2_TiCl_6_	Nb_2_O_5_	CdS	ZnSe	MZO
Thickness (nm)	200	1000	100	50	70	150
Bandgap, *E*g (eV)	3.5	2.23	3.46	2.4	2.81	3.3
Electron affinity, Χ (eV)	4.00	4	4.33	4.18	4.09	4
Relative dielectric permittivity, εr	9.00	19	45	10	8.6	66
Conduction band effective density of states *N*_C_ (1/cm^3^)	2.2$\:\times\:$10^18^	1$\:\times\:$10^19^	1.0 × 10^19^	2.2$\:\times\:$10^18^	2.2$\:\times\:$10^18^	2.2$\:\times\:$10^18^
Valence band effective density of states *N*_V_ (1/cm^3^)	1.8$\:\times\:$10^19^	1$\:\times\:$10^19^	2.2$\:\times\:$10^18^	1.9$\:\times\:$10^19^	1.8$\:\times\:$10^18^	1.8$\:\times\:$10^19^
Electron thermal velocity (cm s^−1^)	10^7^	10^7^	10^7^	10^7^	10^7^	10^7^
Hole thermal velocity (cm s^−1^)	10^7^	10^7^	10^7^	10^7^	10^7^	10^7^
Electron mobility, µn (cm^2^/Vs)	20	4.4	20.73	100	4$\:\times\:$10^2^	100
Hole mobility, µh (cm^2^/Vs)	10	2.5	20.73	25	1.1$\:\times\:$10^2^	25
Donor density, *N*_D_ (1/cm^3^)	10^18^	10^19^	1$\:\times\:$10^15^	1$\:\times\:$10^18^	1$\:\times\:$10^18^	1$\:\times\:$10^18^
Acceptor density, *N*_A_ (1/cm^3^)	0	10^19^	0	0	0	0
Total density (cm^−3^)	10^15^	10^14^	10^15^	1$\:\times\:$10^15^	1$\:\times\:$10^15^	10^15^
References	^63^	^4^ ^[,﻿64^	^32^ ^[,40^	^64–67^	^34^ ^[,68^ ^[,69^	^70^

**Table 2:** Features of htls.

Material property	ZnTe	CNTS	MoO_3_	CuAlO_2_	CdTe	C_6_TBTAPH_2_	nPB	TiO_2_:*N*	NiCo_2_O_4_	D-PBTTT-14
Thickness (nm)	250	100	100	350	200	120	50	50	70	100
Bandgap, *E*g (eV)	2.25	1.74	3.0	3.46	1.5	1.59	2.4	3	2.3	2.16
Electron affinity, Χ (eV)	3.73	3.87	2.3	2.5	3.9	3.58	3	2.2	3.48	3.2
Relative dielectric permittivity, εr	7.3	9	18	60	9.4	3	3	3	11.9	10
Conduction band effective density of states *N*_C_ (1/cm^3^)	2.2$\:\times\:$10^18^	2.2$\:\times\:$10^18^	1$\:\times\:$10^19^	2.2$\:\times\:$10^18^	8$\:\times\:$10^17^	1.3$\:\times\:$10^18^	1$\:\times\:$10^21^	1.3$\:\times\:$10^14^	2.2$\:\times\:$10^18^	2.8$\:\times\:$10^19^
Valence band effective density of states *N*_V_ (1/cm^3^)	1.8$\:\times\:$10^19^	1.8$\:\times\:$10^19^	2.2$\:\times\:$10^18^	1.8$\:\times\:$10^19^	1.8$\:\times\:$10^19^	5.3$\:\times\:$10^18^	1$\:\times\:$10^21^	1.3$\:\times\:$10^15^	1$\:\times\:$10^19^	1.0$\:\times\:$10^19^
Electron thermal velocity (cm s^−1^)	10^7^	10^7^	10^7^	10^7^	10^7^	10^7^	10^7^	10^7^	10^7^	10^7^
Hole thermal velocity (cm s^−1^)	10^7^	10^7^	10^7^	10^7^	10^7^	10^7^	10^7^	10^7^	10^7^	10^7^
Electron mobility, µn (cm^2^/Vs)	300	11	210	2	3.2$\:\times\:$10^2^	0.17	6.1$\:\times\:$10^−6^	2	1.05	2.83$\:\times\:$10^−3^
Hole mobility, µh (cm^2^/Vs)	100	11	210	8.6	4$\:\times\:$10^1^	0.17	6.1$\:\times\:$10^−4^	2	1.61	2.83$\:\times\:$10^−3^
Donor density, *N*_D_ (1/cm^3^)			0	0	0	0	0	0	0	0
Acceptor density, *N*_A_ (1/cm^3^)	1.0$\:\times\:$10^16^	1.0$\:\times\:$10^19^	1$\:\times\:$10^18^	3$\:\times\:$10^18^	2.0$\:\times\:$10^14^	2.2$\:\times\:$10^18^	1$\:\times\:$10^16^	1.3$\:\times\:$10^14^	1$\:\times\:$10^18^	1$\:\times\:$10^18^
Total density (cm^−3^)	1$\:\times\:$10^14^	1$\:\times\:$10^14^	1$\:\times\:$10^15^	1$\:\times\:$10^15^	1$\:\times\:$10^15^	1$\:\times\:$10^17^	1$\:\times\:$10^15^	1$\:\times\:$10^15^	1$\:\times\:$10^15^	1$\:\times\:$10^14^
References	^35^	^36^	^32^,^59^,^71^	^34^,^72^,^73^	^37^,^74^	^38^	^75^	^30^	^76^	^33^

**Table 3:** Input parameters of interface defect layers^77^.

Interface	Defect type	Capture Cross Section: Electrons/holes (cm^2^)	Energetic Distribution	Reference for defect energy level	Total density (cm^−2^) (integrated over all energies)
ETL/Cs_2_TiCl_6_	Neutral	1.0 × 10^−17^ 1.0 × 10^−18^	Single	Above the VB maximum	1.0 × 10^10^
Cs_2_TiCl_6_/HTL	Neutral	1.0 × 10^−18^ 1.0 × 10^−19^	Single	Above the VB maximum	1.0 × 10^10^

##  Result and discussion

### Effect of HTL variation

**Fig. 2:**
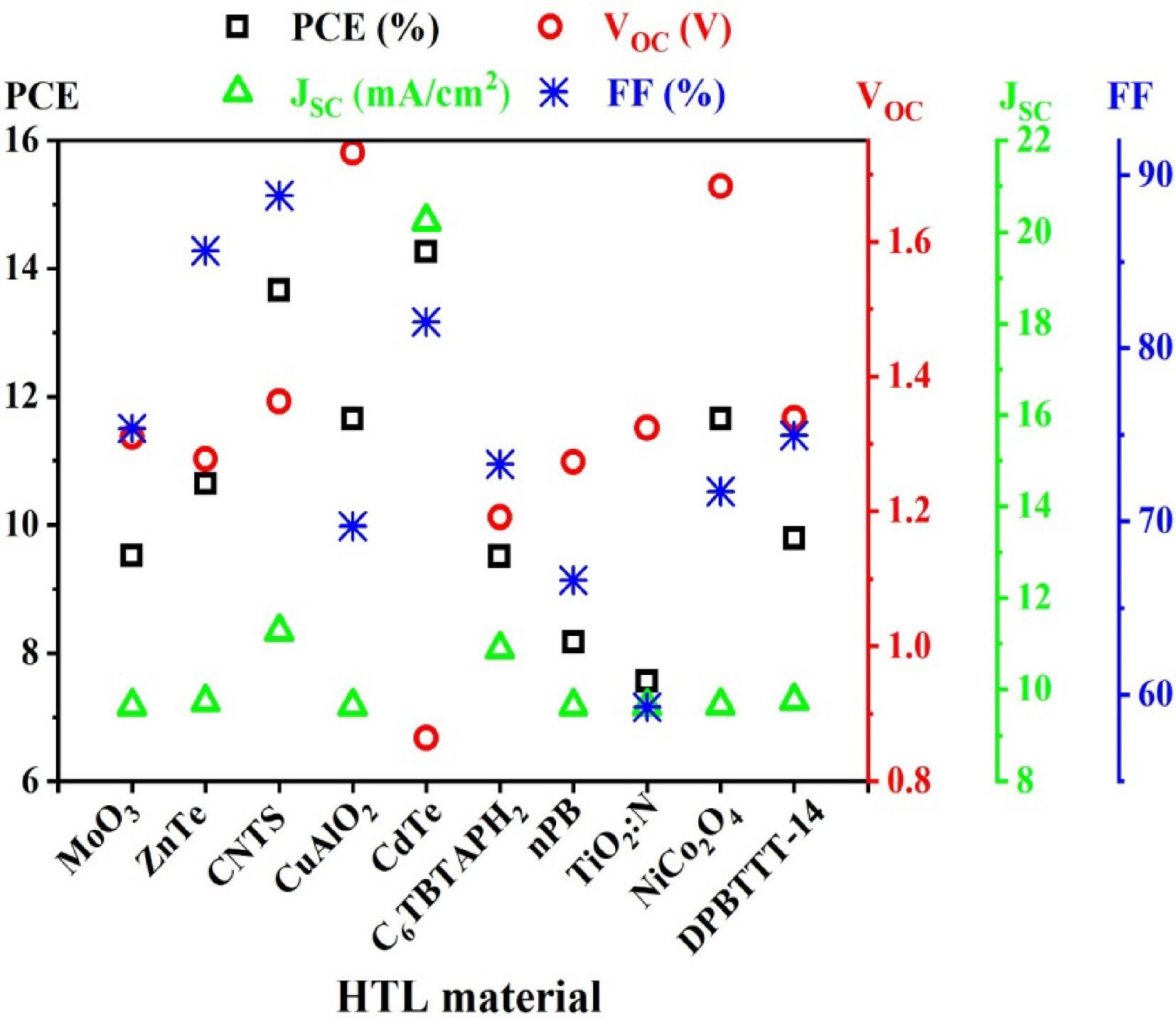
Impact of different HTLs on PSC parameters when CdS is used as ETL.

The hole transport material acts as a compelling element for the successful reversal of oxidation states between the conducting hole and light absorber in PSCs. In addition, the physical location change of holes from the absorber layer to rear contact is assisted by the HTL in Perovskite cell configurations^78,79^. To interpret the influence of HTL on the PV parameters of the device, 10 HTLs such as MoO_3_, ZnTe, CNTS, CuAlO_2_, CdTe, nPB, C_6_TBTAPH_2_, N: TiO_2_, NiCo_2_O_4_, and PBTTT-C14 are utilized considering CdS as ETL. The PV features for instance PCE, *V*_OC_, FF, and *J*_SC_ are illustrated in Fig. 2 in this study.

The highest percentage of FF (77%) is observed by CNTS as opposed to the lowest, 60% is accounted for by N: TiO_2_. The remaining HTLs show the range of 67–85% FF throughout the simulation. In terms of *V*_OC,_ around 0.87 V is obtained by CdTe which is the minimal in this particular device. The surprising fact is that MoO_3,_ N: TiO_2;_ and PBTTT-C14 display the same voltage of about 1.35 V while CuAlO_2_ has shown a superior voltage at 1.79 V. On the other hand, the maximal short circuit current has been recorded for CdTe, at 20 mA/cm^2^, whereas 4 HTLs, for instance, MoO_3_, ZnTe, CuAlO_2_, nPB, NiCo_2_O_4_, and PBTTT-C14 are exhibited the least of *J*_SC_ e.g., 0.9 mA/cm^2^. The remaining two HTLs, C_6_TBTAPH_2_ and CNTS have reported 1.0 mA/cm^2^ and 1.2 mA/cm^2^, respectively. As regards PCE, CdTe has notched up 14.5% of PCE and contrarily the minimum PCE (7.3%) has accounted for the N: TiO_2_. The rest ratio of PCE is altered between 8% and 13.8% to the residual HTLs.

###  Device optimization

#### Optimization of absorber layer thickness

**Fig. 3:**
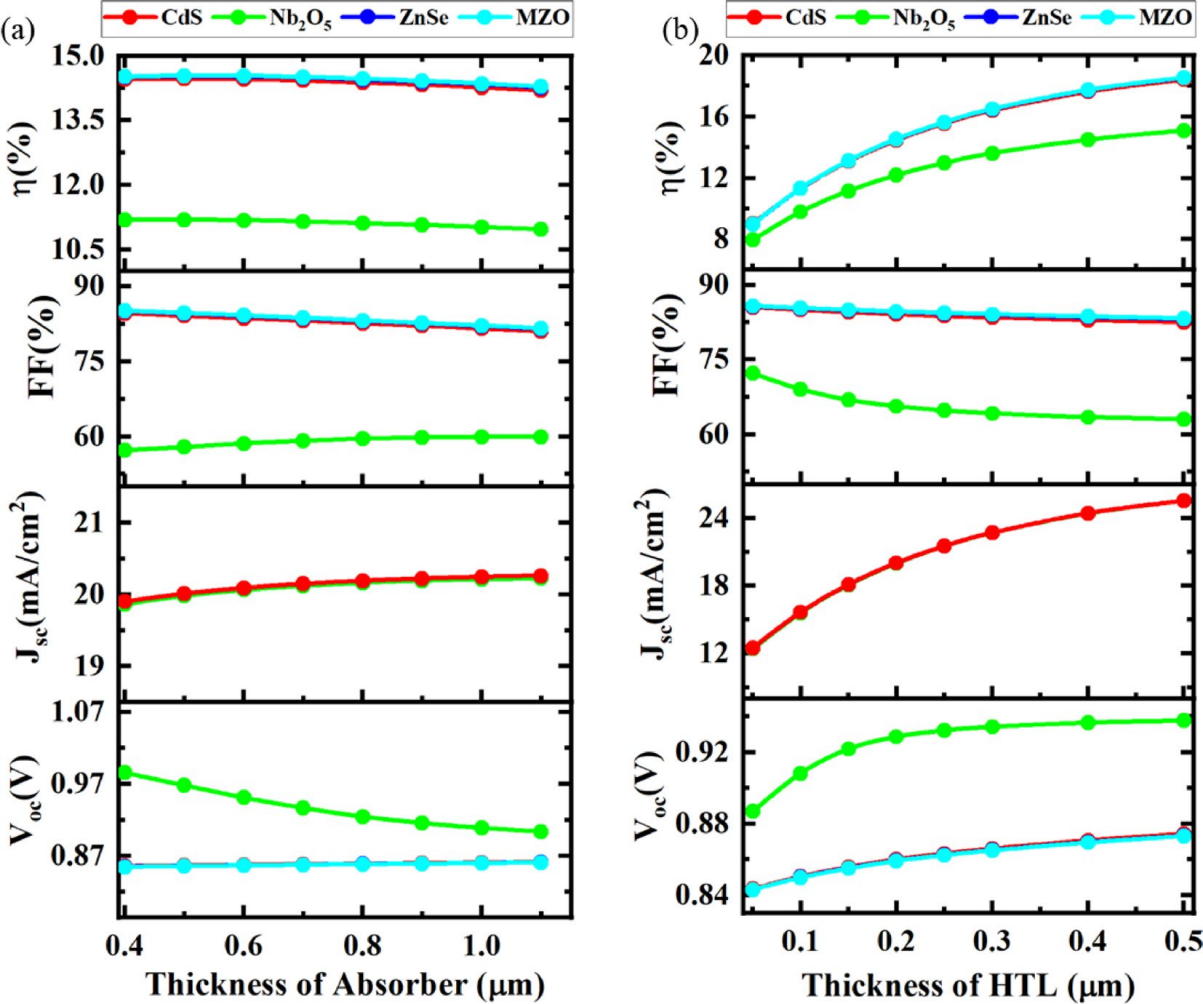
Influence of thickness of absorber **a** and HTL **b** on PSC device performance.

The absorber layer thickness plays a noteworthy role in inducing the lifetimes and diffusion lengths of generated charge carriers^80,81^. To stimulate the effect on PV constraints, the absorber layer thickness differed from 0.4 μm to 1.15 μm, while the thickness of ETL and HTL remained unaffected as represented in Fig. 3a

For Nb_2_O_5_-based PSC, when the thickness was enhanced, the *V*_OC_ declined from 0.98 V to 0.91 V and the FF and *J*_SC_ experienced a slight rise by 1.5% (in magnitude) and by 0.2 mA/cm^2^. In addition, the SC efficiency did not show any changes but remained unchanged as absorber thickness increased.

For CdS-based devices, on the other hand, the *V*_OC_, and *J*_SC_ increased very negligibly while the FF and PCE decreased barely with the enhancement of the absorber layer. Similarly, ZnSe and MZO-based SC configurations also showed homogeneous graphs for PV parameters when the absorber layer was mostly thickened. So, the ideal absorber layer thickness was obtained to be 500 nm in this study in order to gain the superior performance of each device. This value was implemented for the later parts of this research.

####  Optimization of HTL layer thickness

The significance of HTL layer thickness upon different PV parameters is represented in Fig. 3b when the thickness ranges from 10 nm to 500 nm. To figure out the optimum thickness of HTL, the ETL thickness and optimized absorber thickness were fixed. As the HTL was increased, the *V*_OC_ for Nb_2_O_5_-based device surged to 0.94 V compared to 0.86 V for CdS, ZnSe, and MZO-based structures, the *J*_SC_ of all devices escalated considerably from 12 mA/cm^2^ to 26 mA/cm^2^. With regards to FF, there was a slight plunge (by 7%) to the Nb_2_O_5_ device from 10 nm to 200 nm HTL thickness, then it remained stable. However, the remaining three devices corresponded that there was no reliance on the thickness of HTL because the graph was literally flat. However, when the thickness of HTL enlarged, the PCE of CdS, ZnSe, and MZO-based devices jumped to 18% (in magnitude) in comparison with the Nb_2_O_5_ device (14%). A thinner HTL may not provide complete coverage of the absorber layer, leading to increased recombination at the interface. As the thickness increases, hole extraction becomes more efficient, improving Voc and FF. However, excessive thickness results in higher resistive losses, thereby reducing the overall efficiency. In this circumstance, 200 nm was chosen to be the optimum HTL thickness which was followed for the next phases of this study. Increasing the thickness initially enhances the electron extraction and transport, thereby reducing recombination at the interface. However, beyond an optimal thickness, the series resistance increases, which limits current flow and reduces both the short-circuit current (Jsc) and fill factor (FF). Due to the purpose of avoiding recombination of charge carriers, the thickness of ETL should be less than the thickness of HTL^82^.

#### Influence of absorber and HTL thickness on cell performance

**Fig. 4:**
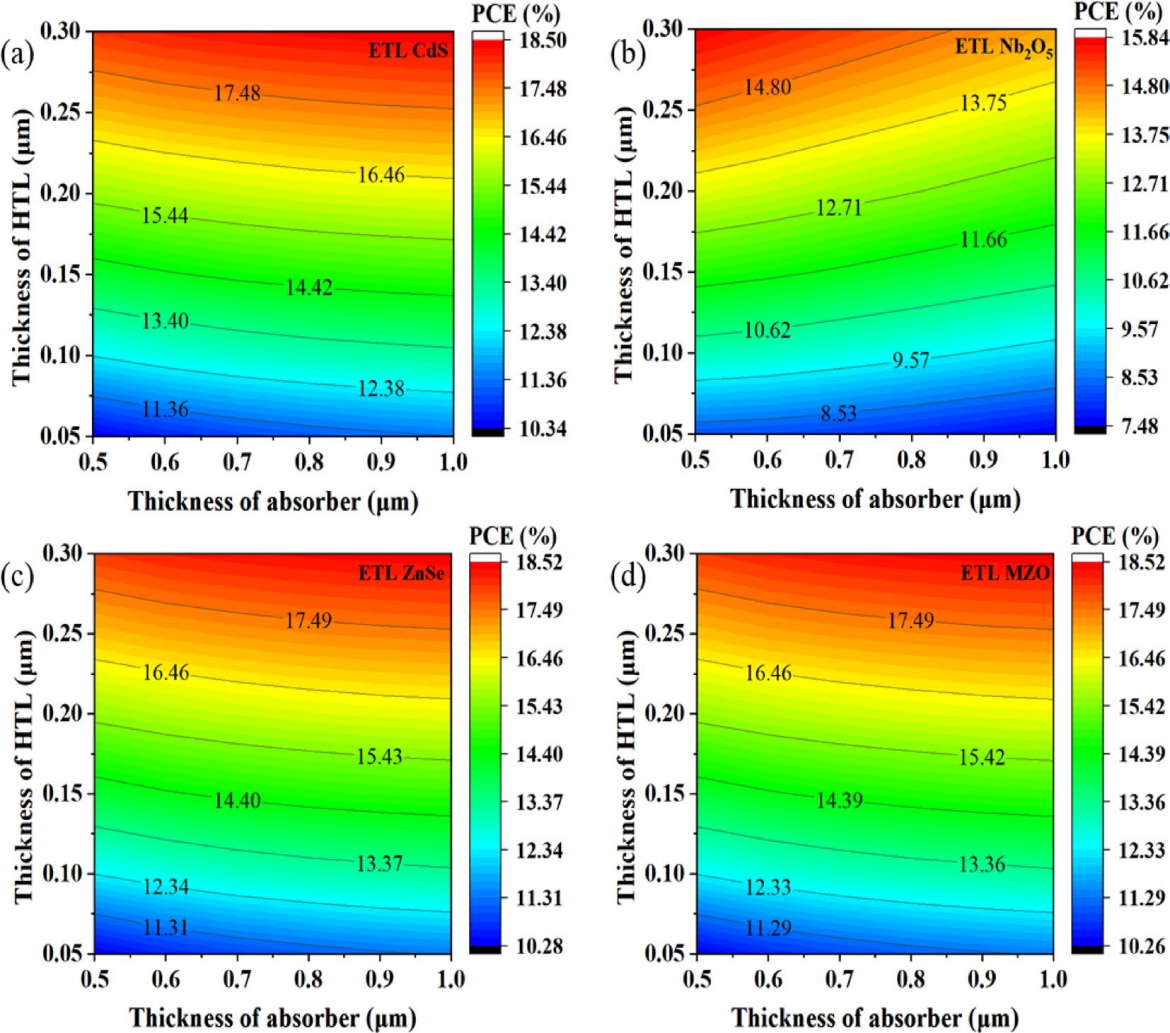
Contour mapping of PCE with respect to the thickness of the Cs_2_TiCl_6_ and CdTe HTL.

The contour diagram Fig. 4 depicts the evaluation of the cell efficiency by simultaneously modulating the absorber thickness and CdTe HTL thickness of Cs_2_TiCl_6_-based PSC with four different ETLs. According to Fig. 4a, 16.46% PCE was obtained for CdS ETL used PSC when the thickness of absorber and CdTe HTL were from 0.5 to 1.0 μm and 0.23 to 0.30 μm, respectively. However, Nb_2_O_5_ was taken as ETL; the device showed in Fig. 4b the lowest 13.75% PCE, since changing the absorber thickness up to 1.0 μm, the thickness of CdTe HTL was ≥ 0.22 μm. It was observed in Fig. 4c that the ZnSe-associated SC and the device configuration with MZO as ETL in Fig. 4d documented the exact same percentage of PCE (16.46%) as the CdS-based device showed when increasing the thickness of absorber ≥ 0.5 μm and thickness of CdTe HTL was greater than 0.23 μm.

####  Influence of absorber defect density and acceptor density on PCE

**Fig. 5:**
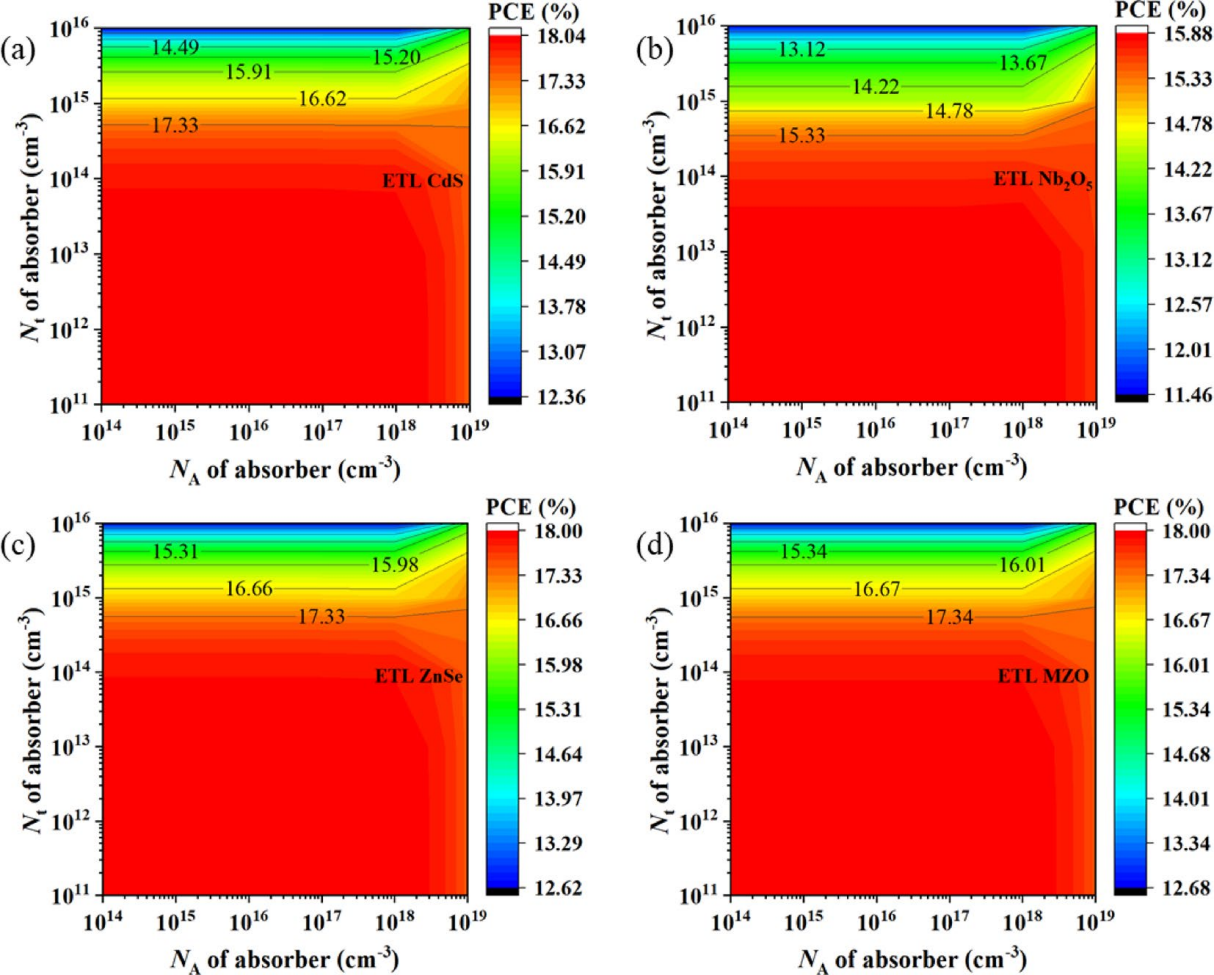
Contour mapping of PCE with respect to varying absorber defect density (*N*_t_) and acceptor density (*N*_A_) for **a** CdS, **b** Nb_2_O_5_, **c** ZnSe, and **d **MZO ETL configured devices.

The change of perovskite cell efficiency by varying the acceptor density (*N*_A_) and defect density (*N*_t_) of the absorber has been demonstrated for four studied structures in Fig. 5. When CdS was used as ETL shown in Fig. 5a, the structure had 16.62% PCE with *N*_A_ fluctuating between 10^14^ cm^−3^ and 10^19^ cm^−3^ and *N*_t_ was increased till 10^15^ cm^−3^. As indicated by Fig. 5b, among all configured devices, Nb_2_O_5_ ETL accounted for the lowest PCE of 14.78% when acceptor concentration was 10^14^ ≤ *N*_A_ ≥ 10^19^ cm^−3^ but defect density (*N*_t_) went up to less than 10^15^ cm^−3^.

In addition, 16.66% SC efficiency was recorded in Fig. 5c for devices with ZnSe ETL while *N*_t_ was increased just above 10^15^ cm^−3^ but *N*_A_ oscillated from 10^14^ to 10^19^ cm^−3^. However, in comparison with other configurations, the PSC with MZO ETL Fig. 5d witnessed the highest efficiency of 16.67% as long as the *N*_t_ and *N*_A_ of the absorber intensified up to 10^15^ cm^−3^, between 10^14^ and 10^19^ cm^−3^, respectively. Since the PV performance of PSC together depends on PCE, FF, *V*_OC_, and *J*_SC_, the increasing value of *N*_t_ and *N*_A_ raised the efficiency performance to a certain limit upon the selection of absorber and HTL material which is relevant to the previous study ^83^.

#### Band alignment

**Fig. 6:**
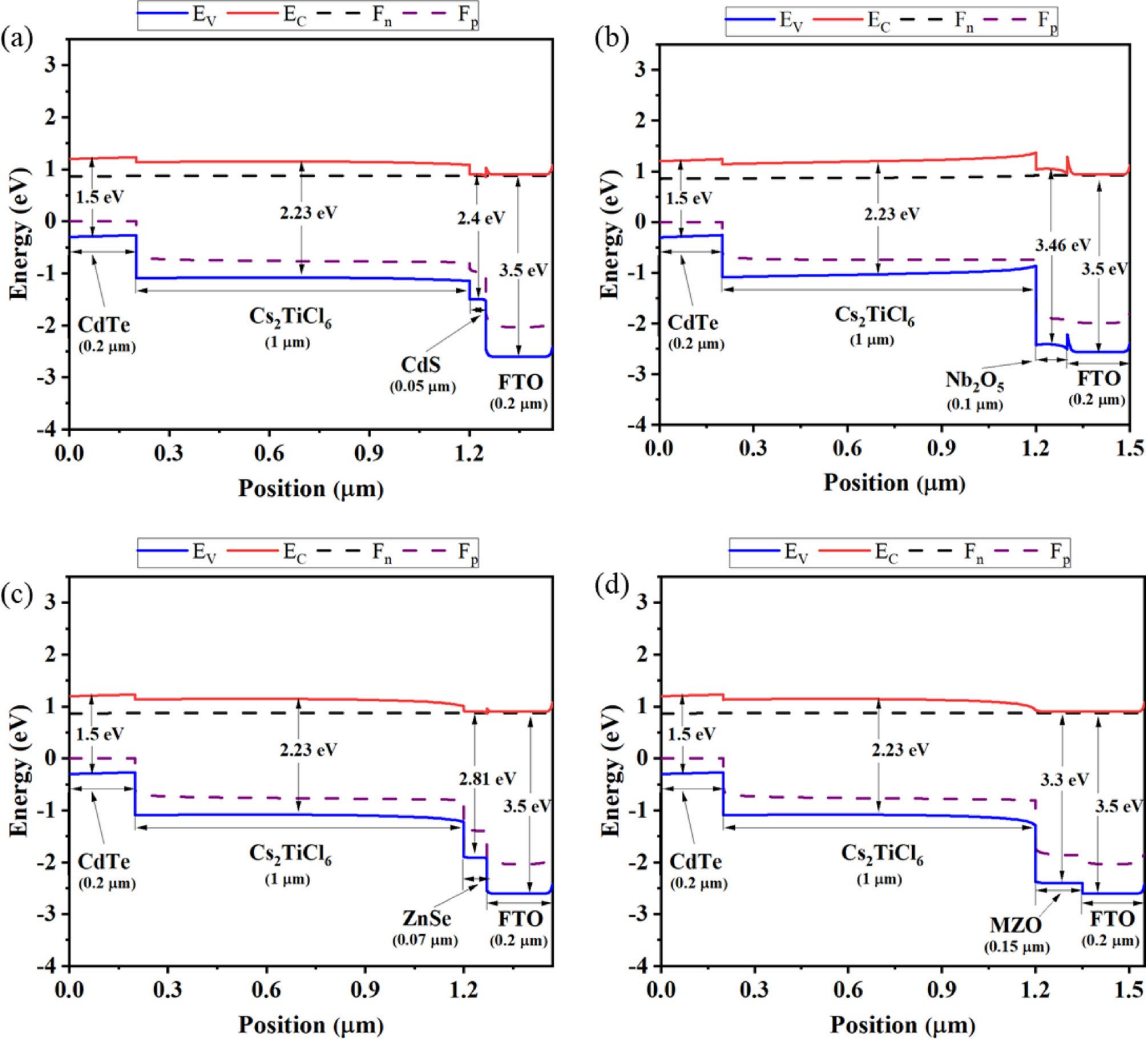
Energy band diagram of Cs-based PSCs with CdTe as the HTL and **a** CdS, **b **Nb_2_O_5_, **c** ZnSe, and **d** MZO as ETL.

The alignment of energy levels exhibits a substantial impact on the performance and effectiveness of PSC. It has been developed using each ETL paring with Cs_2_TiCl_6_ absorbing layer and CdTe as HTL that enhances influence on the difference in valence band (VB) levels between the HTL and the absorber layer and the conduction band (CB) levels between the ETL and the absorber layer. Within the framework of PSCs, photo-excited electrons are concurrently driven into the CB of the ETL, while positively charged holes make their way to the HTL. Afterward, electrons and holes converge at the front layer (FTO) and back (Au) contact metals, individually. To ensure the safe extraction of electrons at the interface between ETL and Cs_2_TiCl_6_, the ETL requires an increased electron affinity compared to Cs_2_TiCl_6_. Simultaneously, the ionization energy of the HTL must be lower than that of Cs_2_TiCl_6_ for efficient hole extraction at the point of contact between Cs_2_TiCl_6_ and the HTL. Furthermore, the unstable energy band at both intersections has a significant influence on the PV parameters of the device. In Fig. 6, quasi-fermi levels (*F*_n_ and *F*_p_) are combined with CB energy (*E*_C_) and VB energy (*E*_V_), respectively. The *F*_P_ was placed at a high up of *E*_V_ in every ETL while *F*_n_ and *E*_C_ are comparatively proportionate to each other. The band gap of Cs_2_TiCl_6_ was 2.33 eV while the band gaps of CdS, Nb_2_O_5_, ZnSe, and MZO were 2.4 eV, 3.46 eV, 2.81 eV, and 3.3 eV, respectively. Conversely, comparatively minimal performance has been found for CdS and ZnSe ETLs because of their corresponding band gaps. CdS, ZnSe, and MZO exhibit better conduction band alignment with the Cs_2_TiCl_6_ absorber layer, which facilitates efficient electron extraction and suppresses interfacial recombination. In contrast, Nb_2_O_5_ has a deeper conduction band minimum that creates a larger offset with the absorber’s conduction band, resulting in a less favorable energy alignment for electron transport.

####  Optimization of acceptor density of the absorber

**Fig. 7:**
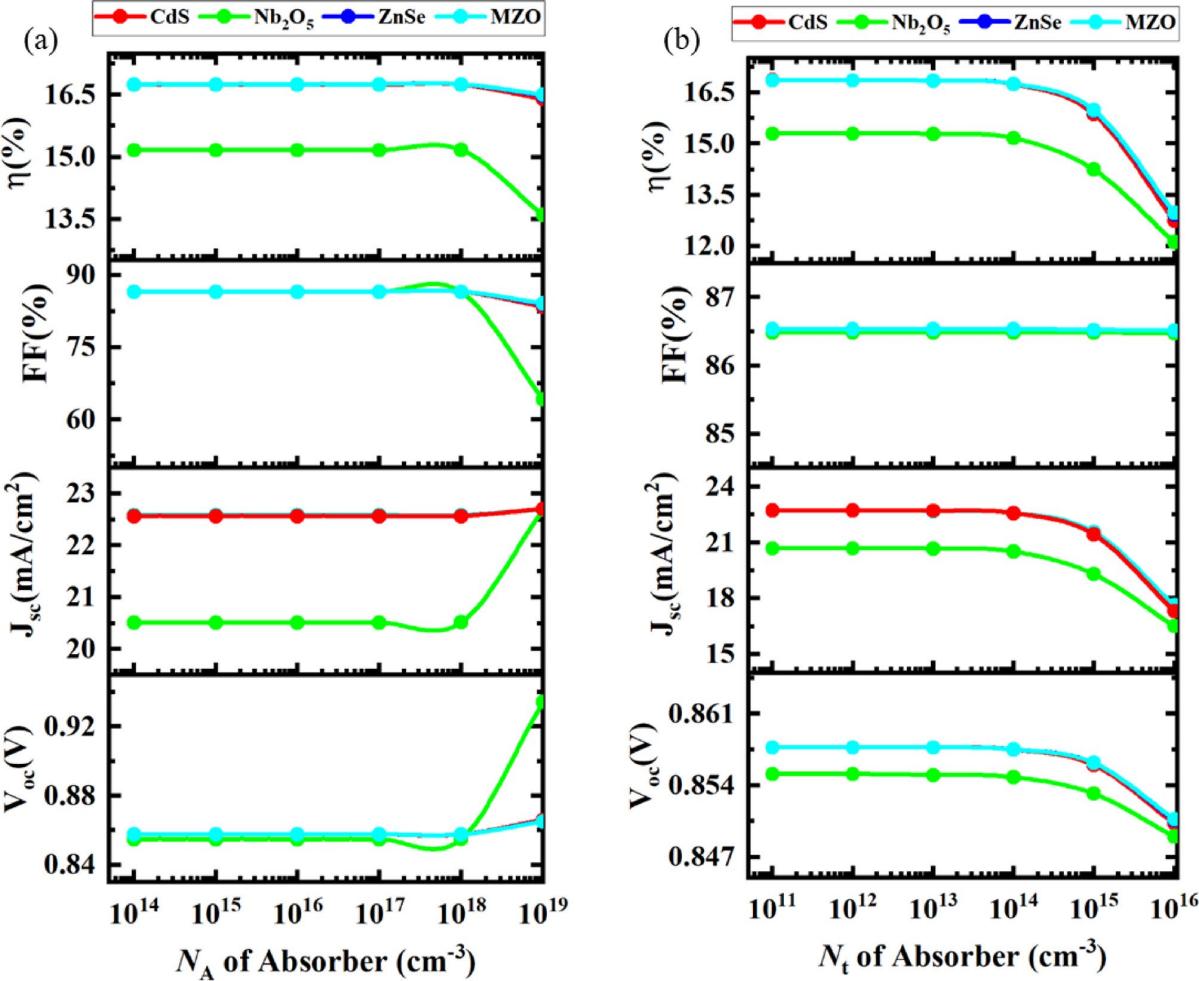
Trends in device performance (PCE, FF, *V*_OC_, and *J*_SC_) with absorber acceptor density and defect density.

For the purpose of gathering meaningful insights into the impact of absorber acceptor density (*N*_A_) on PV parameters, the *N*_A_ of Cs_2_TiCl_6_ for four optimized devices was altered, ranging from 10^14^ cm^−3^ to 10^19^ cm^−3^ as represented in Fig. 7a. It has summarized how the PCE, FF, *V*_OC_, and *J*_SC_ were modulated with the variance of *N*_A_ while other variables of the absorber layer were considered constant.

To begin with, there was not any distinguishable change in all PV features when the *N*_A_ was less than 10^18^ cm^−3 84^. That implies the unchanging direction for the generation rate of photo-generated carriers with *N*_A_ when same the number of photons is accrued ^55,85^. For increasing the acceptor concentration of the Cs_2_TiCl_6_ layer, afterward, the *J*_SC_ remained unchanged for all of the Cs_2_TiCl_6_-based SCs except Nb_2_O_5_-based PSC which had an abrupt increase to 22.5%. As regards *V*_OC_, a similar trend to *J*_SC,_ from 0.86 V to 0.94 V, was observed for Nb_2_O_5_ designed PSC; the other PSCs maintained their respective initial values as *N*_A_ increased. The reason behind the *V*_OC_ rise is due to the fall of the fermi energy level of the hole and the increase of built-integrated potential^84^. When the *N*_A_ value of the absorber further extended above 10^18^ cm^−3^, Nb_2_O_5_-based SCs accumulated a sudden decline from 15 to 13.5% (in magnitude) for cell efficiency (η) and 23% (in magnitude) downturned for FF. As a result, to acquire better outcomes for each combination, the optimum value of *N*_A_ of Cs_2_TiCl_6_ was selected as 10^16^ cm^−3^ which was utilized next periods of this simulation.

#### Optimization of defect density of the absorber

The illustration in Fig. 7b shows how the variation of defect density of the absorber (*N*_t_) has influenced the device performance. The *N*_t_ of the Cs_2_TiCl_6_ absorber layer was inspected for selected structures on the scale of 10^14^ cm^−3^ to 10^19^ cm^−3^. With the increasing *N*_t_ value, there was a deterioration in SC features like η, FF, *V*_OC_, and *J*_SC_. As reported by this observation, the values of *V*_OC_, *J*_SC,_ and PCE got stable till the *N*_t_ attained at 10^15^ cm^−3^ but the FF remained uniform at 10^19^ cm^−3^ of defect density for all optimized configurations. Then *N*_t_ of 10^15^ cm^−3^ onwards, *V*_OC_, *J*_SC_, and PCE displayed a similar kind of decrement such as from 0.855 V to 0.848 V, from 21 mA/cm^2^ to 16 mA/cm^2^, also from 15.5 to 12%, respectively when Nb_2_O_5_ was installed as ETL. Conversely, CdS, ZnSe, and MZO-based SCs exhibited maximum PCEs of 17.6% on average (in magnitude) at their individual best *N*_t_. Ultimately at 10^19^ cm^−3^ of *N*_t_, the distinct cell efficiency was reduced to 12.7% on average. The declined efficiency of the structures was subjected to the non-radiative Shockley-Read-Hall (SRH) recombination, thus causing diminishing carrier lifetimes, increased recombination rates, and a substantial reduction in the overall performance of the device ^86^. Decreasing *N*_t_ provides the best PCE, but it can be ignored as optimum value because it is a daunting task to integrate a material experimentally^84^. These findings were nearly commensurable to the previously published research work^87^. Therefore, the optimum *N*_t_ of the Cs_2_TiCl_6_ absorber was taken as 10^13^ cm^−3^. This value was maintained in the following steps of this study. A significant body of experimental data suggests that polycrystalline thin films of lead halide perovskites usually demonstrate defect densities around 10^15^–10^16^ cm^−3^ while single crystals generally show bulk defect densities of 10^12^ cm^−3^ or lower ^88,89^. In this study, the defect density of the perovskite absorber has been varied from 10^14^ cm^−3^ to 10^19^ cm^−3^ and the optimized defect density of the device was found to be 10^13^ cm^−3^, which aligns well with experimentally reported values for perovskite solar cells. For example, Heo et al. demonstrated a device efficiency of 13.5% with CH_3_NH_3_PbI_3_ having a defect density of 1.3 × 1015 cm^−3 90^. Another study reported trap densities of 5.65 × 10^15^ and 2.25 × 10^15^ cm^−3^ where the lowest trap density (2.25 × 10^15^ cm^−3^) corresponded to the highest PCE ^91^. These comparisons confirm that our defect density is within a realistic and competitive range for efficient solar cell operation.

#### Optimization of donor density of ETL

**Fig. 8:**
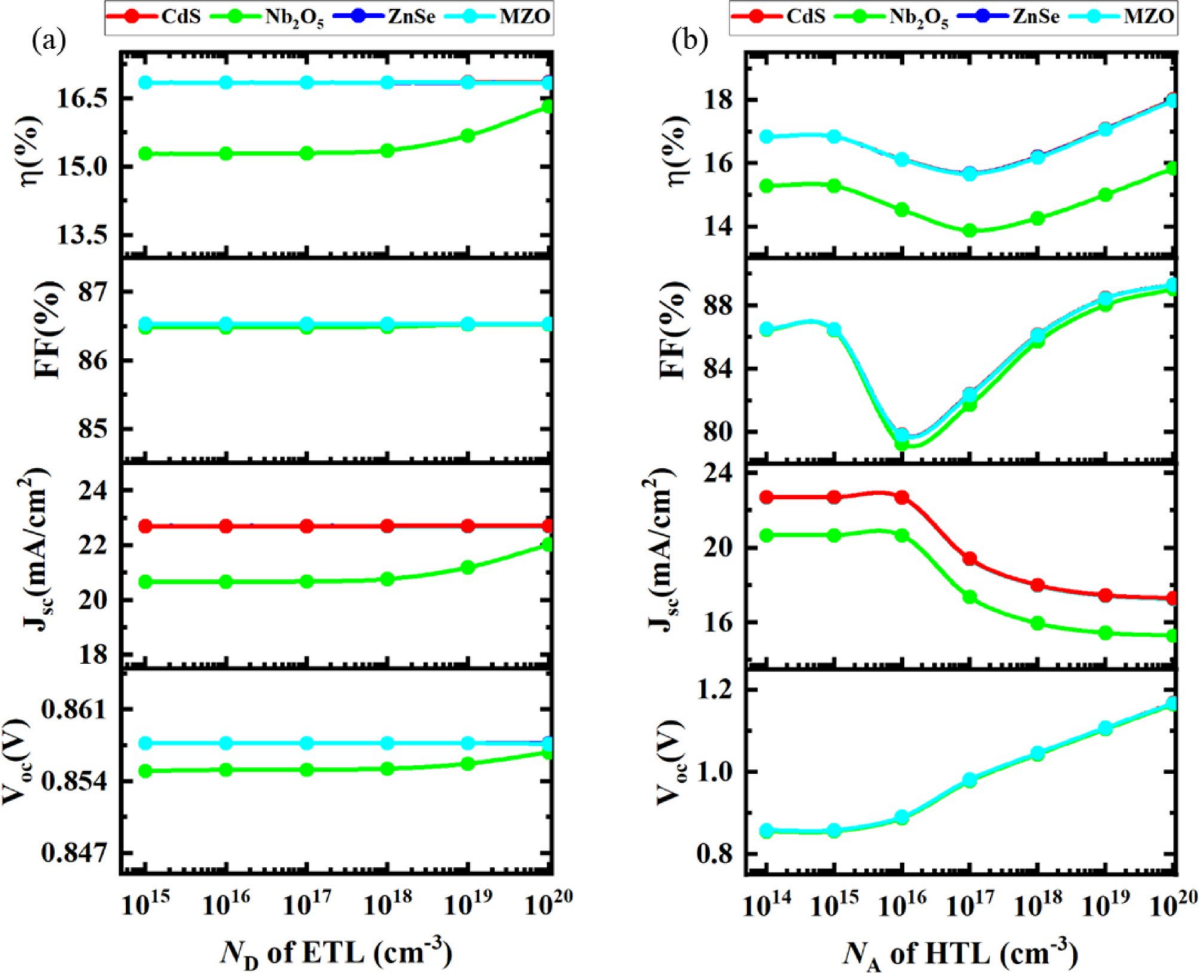
Analysis of **a** donor density (*N*_D_) of ETL, and **b** acceptor density (*N*_A_) of HTL.

The representation of Fig. 8a states the modulation of PCE, FF, *V*_OC_, and *J*_SC_ with respect to changing the donor density (*N*_D_) of ETL. To gain better effectiveness, the *N*_D_ of ETL was adjusted within the range of 10^15^ cm^−3^ to 10^20^ cm^−3^. In this investigation for all device structures, it is evident that FF was stable all through the variation of donor density of ETL. Whereas, the *V*_OC_ encountered different patterns among all executed ETLs where CdS, ZnSe, and MZO-installed devices got unaltered apart from Nb_2_O_5_–based PSC that had a slight increase between *N*_D_ of 10^18^ cm^−3^ to 10^20^ cm^−3^. Moreover, up to 10^18^ cm^−3^, *J*_SC_ and PCE remained almost consistent for PSC when Nb_2_O_5_ was ETL, later they tended to rise from 20.8 mA/cm^2^ to 22 mA/cm^2^ and from 15.3 to 16.5% (in magnitude), respectively, as opposed to other optimized devices with CdS, ZnSe and MZO combinations kept static throughout the period of increasing *N*_D_ of ETL. The higher value of *N*_D_ of ETL provides the assistance of charge extracting and carrying them out to the ETL/absorber layer. Contrarily, series resistances can the PV features low at a decreasing value of *N*_D_^92,93^. The same kind of trends were also attained in published findings^94^. To find better-optimized results for each device, the optimal donor density of ETL was considered as 10^18^ cm^−3^ which was exactly the same as the initial value.

####  Optimization of acceptor density of HTL

The illustration describes the effect of the acceptor density of HTL on the cell performance of Cs_2_TiCl_6_-based PSCs shown in Fig. 8b The variation of acceptor density (*N*_A_) has been measured from the limit of 10^14^ cm^−3^ to 10^20^ cm^−3^, concurrently holding the other performance parameters constant for all optimized devices.

While the *N*_A_ was increased, the *V*_OC_ was static for a while before initiating the steep rise to 1.2 V for all simulated combinations. On the subject of *J*_SC_, each device has maintained a similar pattern of stability till 10^14^ cm^−3^ of acceptor density, then decreasing to 15.8 mA/cm^2^ for PSC using Nb_2_O_5_ as ETL in contrast declined to 17.5 mA/cm^2^ accounted to rest of structures. There had been a remarkable fluctuation curve witnessed for FF with respect to the increment of HTL *N*_A_. At first, the FF decreased from 86 to 82% (in magnitude), then the FF curve commenced to burgeon dramatically and reached 89% at 10^20^ cm^−3^ of HTL *N*_A_. With the increase in *N*_A_ of HTL, the cell efficiency started collapsing up to 10^17^ cm^−3^ afterward the efficiency of CdS, ZnSe, and MZO-configured devices escalated to 18% compared to 16% when CdS was implemented as an ETL. As the *V*_OC_ recorded a swift rise along with the growing *N*_A_ of HTL that can be the objective of electric potential in between the intersection of HTL and perovskite absorber ^95^. In contrast, the PCE and FF increased as the *N*_A_ of HTL expanding further facilitates the conductivity which has an astonishing impact on accumulating charges because of the upgraded electric field existing^96^.

###  Optimized J-V and Q-E characteristics

**Fig. 9:**
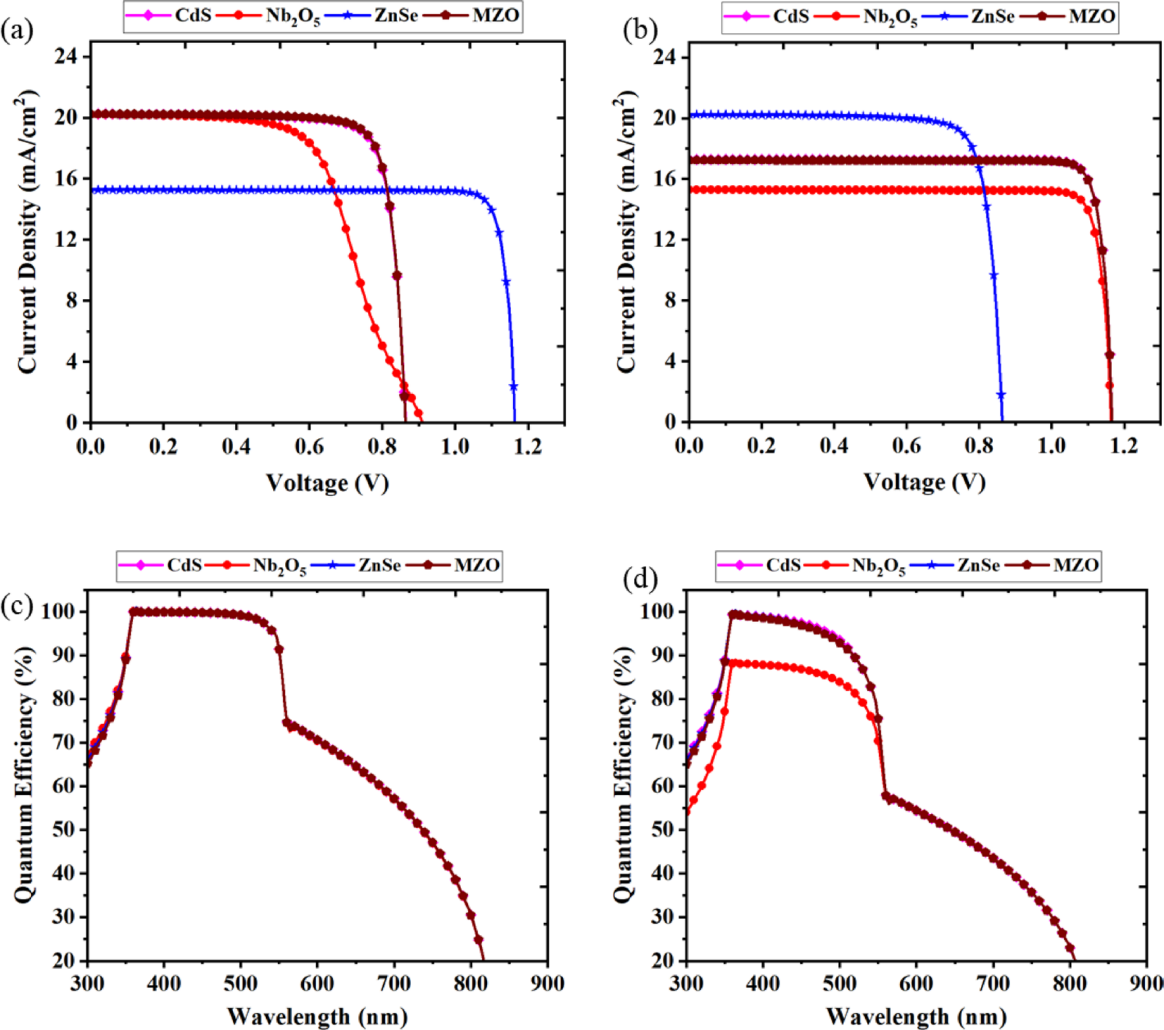
**a** The current density–voltage (J-V) curve for the pristine optimized structure, **b** the current density–voltage (J-V) curve for the final optimized structure, **c** the Q-E curve of the pristine optimized structure and **d** the Q-E curve of the final optimized device.

From Table 1, it is evident that key parameters such as bandgap and electron affinity, which play critical roles in band alignment, are quite similar across the ETL materials studied. Other properties, including electron and hole mobilities, also show comparable values. This similarity in parameter values suggests that these ETL materials exhibit consistent behavior in device performance. A similar trend has also been observed in previous study^13^. The requirement of having fundamental knowledge about short circuit current density with respect to voltage is compelling when the SC gets stimulated. The changes in current density in connection with voltage for pristine and final optimization have been shown in Figs. 9a,b. As the voltage increased, the current densities remained unchanged and had been precipitously shrinking for all optimized structures at some point of increasing voltage. In Fig. 9a, it was observed that ZnSe as an ETL-related device showed *J*_SC_ of about 15 mA/cm^2^ until the *V*_OC_ was ˃1.58 V; concurrently the *J*_SC_ was almost 20 mA/cm^2^ for Nb_2_O_5_, MZO and CdS-associated structures at ˂0.9 V of Voltage. However, at the final stage, all different ETL-configured configurations except the device built with ZnSe prolonged their consistent trend of *J*_SC_ up to *V*_OC_ of less than 1.18 V on increasing voltage in the final optimization process. While the linked device had shortened the *J*_SC_ curve when *V*_OC_ was ˂0.88 V, the value of *J*_SC_ went up to 20 mA/cm^2^. Exhibiting defect states in the absorber layer has a considerable impact on all PV features. The findings matched with previous literature that solid crystallinity impedes charge recombination but enhances performance^97^. This observed 3% variation in performance ratio between the pristine and final optimized devices arises due to the complex interplay of factors beyond the basic J-V relationship. Specifically, while voltage and current density are interdependent, the optimization process alters several properties such as interface quality, defect density, and carrier dynamics. Improvements in these areas reduce recombination losses and enhance charge extraction, thereby slightly shifting the J–V characteristics and the fill factor, leading to the observed variation^98,99^. Such changes reflect real physical improvements in device operation rather than inconsistencies.

On the other hand, the QE curve for the pristine and final optimized configurations was displayed in Figs. 9c,d by changing the wavelength ranging from 300 nm to 900 nm. The classification of QE is the ratio of all charge carriers produced by light to the number of photons that hit the solar cell^100^. During the period of pristine optimization, all devices designed with various ETLs had almost half square-shaped QE curves and experienced almost 100% efficiency in the visible wavelength range of 360–560 nm. The CdS, ZnSe, and MZO-based devices had the taste the best QE of 99.7% when the wavelength was about 390 nm as opposed to 85% at 370 nm to Nb_2_O_5_-associated PSC. The poor performance of Nb_2_O_5_ in quantum efficiency (QE) can be attributed to its relatively lower carrier mobility and potential for forming defect states at the ETL/absorber interface, which increases charge carrier recombination. Additionally, the interfacial contact quality with Cs_2_TiCl_6_ may be suboptimal compared to the other ETLs, thereby impeding charge separation and transport.

###  Effect of resistance and temperature

####  Effect of series and shunt resistance

**Fig. 10:**
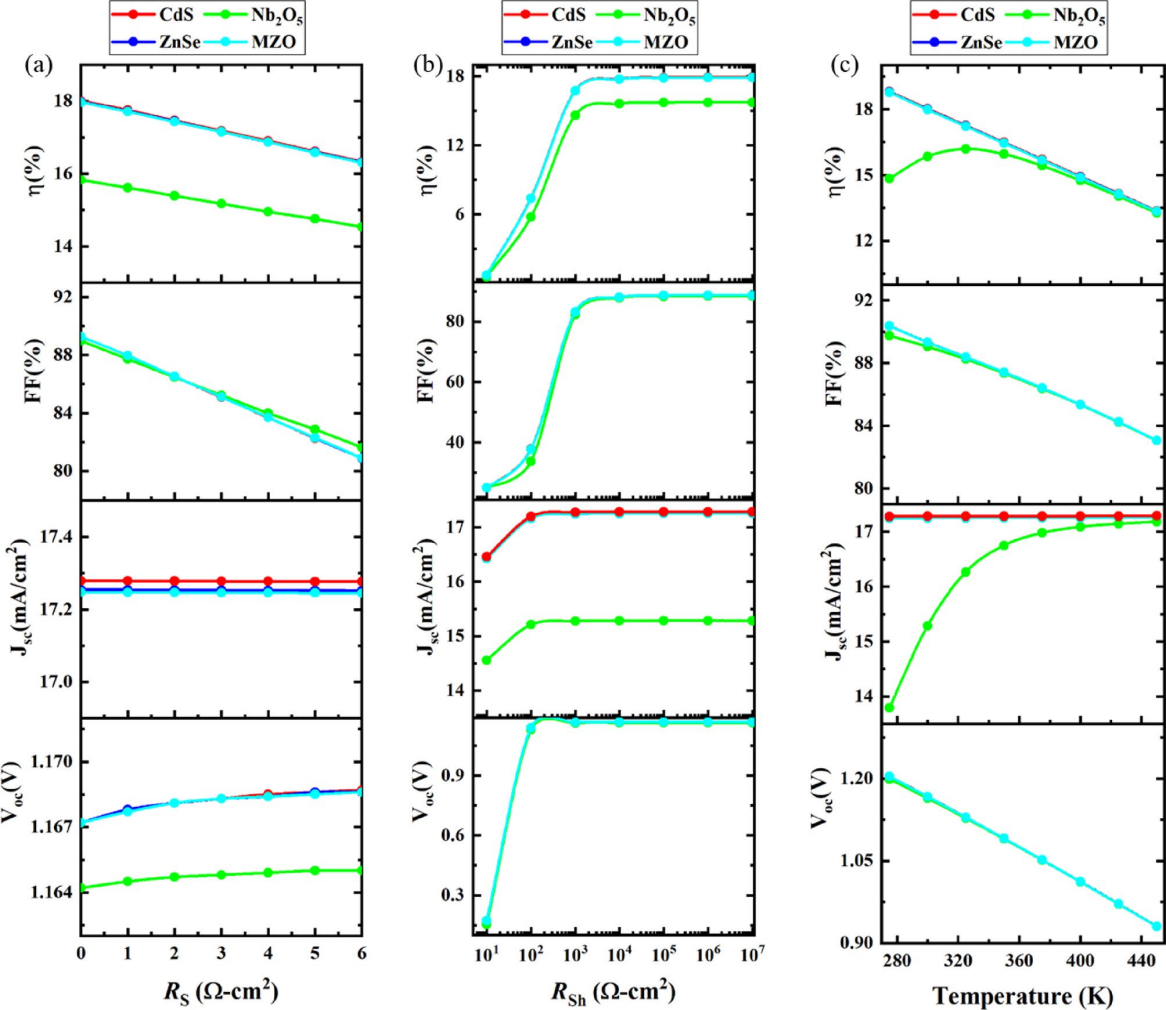
Significance of **a** Series resistance on *V*_OC_ (V), *J*_SC_ (mA cm^−2^), FF (%) and η (%) **b** Shunt resistance on *V*_OC_ (V), *J*_SC_ (mA cm^−2^), FF (%) and η (%). **c** Temperature on *V*_OC_ (V), *J*_SC_ (mA cm^−2^), FF (%) and η (%).

Figure 10a depicts the experimentation of the electrical performance of the series resistance of different PSC structures. In this experiment, series resistance (*R*_S_) is fluctuated between 0$\:\:{\Omega\:}{\text{c}\text{m}}^{2}$ and 6 $\:{\Omega\:}{\text{c}\text{m}}^{2}$, keeping shunt resistance (*R*_Sh_) fixed. When *R*_S_ of optimized cell device increases, there is a marginal rise in the V_OC_ from 1.164 V to 1.165 V for Nb_2_O_5_ -based SCs and from 1.167 V to 1.168 V for CdS, ZnSe and MZO-based PSC. However, increasing series resistance does not influence the *J*_SC_ of SCs. The *J*sc is classified by the ratio of absorbing light by the device to the successful conversion of absorbed photons into electrical current. In SCs, *R*_S_ is caused by the resistance of each layer utilized and by the loss of charge transfer between the layer and metal contacts^101^. However, augmentation of *R*_S_ is considered the main factor for dropping the FF to 82% (in magnitude), and η downturned significantly to an average of 2% (in magnitude) for each SC device. The *R*_S_ is viable for impeding the current flow that leads to power destruction within a device and, eventually, costs the device’s overall performance. So, the best PCE is exhibited when a low series resistance exists.

On the other hand, Fig. 10b shows the alternation of PV parameters along with the shunt resistance for four structures. The routes used for recombination are the cause of shunt resistance^102^. The efficiency of the PSCs is simulated in terms of varying the *R*_Sh_ from 10 $\:{\Omega\:}{\text{c}\text{m}}^{2}$ to 10^7^$\:\:{\Omega\:}{\text{c}\text{m}}^{2}$ when the *R*_S_ stationed constant at 0.5$\:\:{\Omega\:}{-\text{c}\text{m}}^{2}$. Throughout the upswing, *R*_Sh_, the *V*_OC_, and the *J*_SC_ proliferated exponentially to 10^2^$\:\:{\Omega\:}{-\text{c}\text{m}}^{2}$ *R*_Sh_ before remaining unchanged till 10^7^$\:\:{\Omega\:}-{\text{c}\text{m}}^{2}$ for all four optimized devices. On the contrary, between 10 $\:{\Omega\:}{-\text{c}\text{m}}^{2}$ to 10^3^$\:\:{\Omega\:}{-\text{c}\text{m}}^{2}$ *R*_Sh_, the FF, and PCE accounted for a steady increase of 63% (in magnitude) and 15% on average (in magnitude), respectively which is well matched with previous experimental literature^103–105^. After that, both of these continued. The enhancement of PCE is due to the elimination of leakage current which develops when there are some manufacturing defects or imperfections thus raising the *R*_Sh_. A significant *R*_Sh_ lessens the obstruction stated in the p-n junction and permits more current to flow via it^106^.

####  Effect of temperature

The temperature T (K) has a great impact on the performance of PSCs while they are illuminated to sunlight in various geographical regions. So, it is considered an indispensable prerequisite to figuring out the temperature-subordinated electrical parameters for PSC are shown in Fig. 10**(c).** In this study, four different structures are simulated varying the temperature from 275 K to 450 K. For each device, the FF decreased from 90 to 83% and the *V*_OC_ reduced by 30% in voltage respectively with respect to heated temperature. As regards the *J*_SC_, there was an upward trend observed for PSC with Nb_2_O_5_-based PSC due to band gap reduction of material thus more charge pairs will be produced^107^. While the remaining three devices showed stable voltage during that period. Different declinations of cell efficiency have been recorded for all four optimized devices. When the temperature maximized to 450 K, the efficiency of Nb_2_O_5_-based SCs curtailed slowly to 13% when CdS, ZnSe, and MZO-based PSCs followed a similar diminution of cell efficiency promptly to 13%. The reason for deteriorating efficiency can be the stress and deformation are proportioned with temperature increases, therefore, intensifying interfacial defects, causing inadequate internal conduction between layers, and recombination increases as well^108^. Furthermore, the augmentation of temperature is authoritative to the reduction diffusion length (L) which in turn is series resistance, resulting in reduced efficiency^109^. The consistent shrinking of device performance along with rising temperature has been acquired in previous research findings^56^.

###  Analysis of generation and recombination rate

**Fig. 11:**
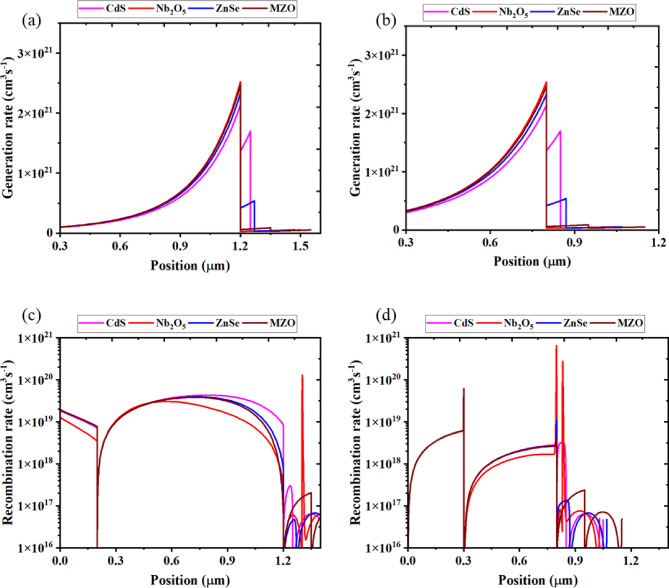
Effect of **a** Generation rate for the initial optimized structure, **b** Generation rate for the final optimized structure, **c** Recombination rate for the initial optimized structure, **d** Recombination rate for the final optimized structure.

The investigation of the influence of generation rate for initial and final optimization has been carried out and demonstrated in Figs. 11a,b along with the changeable position in the device. The transition of excited electrons from the VB to the CB generates the electron-hole pairs, leaving a hole in the VB layer during the carrier production stage which is defined by the release of electrons and holes. For initial optimization in Fig. 11a, the generation rate for all structured devices was 2.5 × 10^21^ cm^3^s^−1^ at 1.2 μm position. That ratio remained unchanged but positioned at 0.8 μm for the final optimization process that was depicted in Fig. 11b. The highest number of electrons produced at that region where the majority of photons are absorbed instigated a significant generation rate in the device. The generation of electron-hole pairs, G (λ, x), can be calculated by SCAPS-1D in terms of the incident photon flux, N_phot_ (λ, x) for each spectrum and region:

$$\:G\:(\lambda\:,\:x)\:=\:\alpha\:\:(\lambda\:\:,\:x).\:Nphot\:(\lambda\:,\:x)\:$$

In contrast, the recombination rate is considered as opposite of the generation rate, involving joining and separating the electrons and holes within the CB. The rate of recombination in perovskite is affected by both the lifetime and density of the charge carrier. Moreover, the imperfection state at each perovskite layer plays a vital impact in electron-hole recombination. During the initial optimization Fig. 11c the recombination rate was almost 1 × 10^19^ cm^3^s^−1^ for all optimized structures Fig. 11d within the range of 0.2 μm to 1.2 μm where CdS showed comparatively the highest recombination. The reason for that maximum rate is more electrons located in the conduction band outstepped the band energy gap and placed into the VB and replaced of place of hole at that limit. Consequently, the electron-hole recombination was affected by energy levels generated at that peak position. Un-even distribution of recombination rates occurs due to the grain boundaries and defects ^110^.

The quantum efficiency (QE) of the devices shows characteristic variation with wavelength. At shorter wavelengths (300–550 nm), the QE is relatively high due to strong absorption near the surface of the absorber, where the photogenerated carriers are effectively collected before recombination. In the longer wavelength range (550–800 nm), the QE gradually decreases because photons penetrate deeper into the device, increasing the likelihood of carrier recombination before collection. The differences in QE among devices using various ETLs arise from differences in conduction band alignment and interface quality, which directly affect carrier extraction. For instance, better band alignment at the ETL/absorber interface leads to improved charge transport and higher QE, whereas poor interface quality or unfavorable band offsets enhance recombination losses, especially for long-wavelength photons

###  Comparison of SCAPS-1D results with previous work

**Table 4:** The comparison of PV constraints of Cs_2_TiCl_6_-based PSCs.

Type	Device structure	V_OC_ (V)	J_SC_ (mA/cm^2^)	FF (%)	PCE (%)	Ref.
1	FTO/TiO_2_/Cs_2_TiBr_6_/P3HT//Au	0.89	3.87	59.5	2.15	^45^
1	FTO/SnO_2_/Cs_2_TiBr_6_/MoO_3_/Au	1.53	8.66	86.45	11.49	^59^
2	FTO/TiO_2_/Cs_2_TiBr_6_/NiO/Au	1.12	10.25	73.59	8.51	^111^
2	FTO/TiO_2_/Cs_2_TiBr_6_/NiCo_2_O_4_/Au	1.34	17.67	82.51	19.30	^76^
2	ITO/NPB/Cs_2_TiBr_6_/PCBM/BCP/Ag	1.29	16.66	78.1	16.85	^75^
2	FTO/TiO_2_/Cs_2_TiBr_6_/CuSCN/Ag	1.90	19.88	35.95	13.57	^112^
2	FTO/TiO_2_/Cs_2_TiCl_6_/CuSCN/Ag	1.98	8.23	33.73	5.50	^112^
2	FTO/CdS/Cs_2_TiCl_6_/CdTe/Au	1.188	17.83	89.51	18.15	^a^
2	FTO/Nb_2_O_5_/Cs_2_TiCl_6_/CdTe/Au	1.186	15.76	89.43	16.07	^a^
2	FTO/ZnSe/Cs_2_TiCl_6_/CdTe/Au	1.183	17.85	89.78	18.03	^a^
2	FTO/MZO/Cs_2_TiCl_6_/CdTe/Au	1.923	17.94	88.98	18.12	^a^

.1- experimental, 2- theoretical, a this work.

The latest experimental and theoretical findings on Cs_2_TiCl_6_-based PSCs are comparatively analyzed with our study and shown in Table 4. The optimized Cs_2_TiCl_6_-based PSC presents a higher PCE compared to the previously used device structure with a similar kind of halide perovskite absorber. The device structures had a desired PCE are 15% while previously device configurations like FTO/TiO_2_/Cs_2_TiCl_6_/CuSCN/Ag obtained 5.5% of PCE. In comparison with other Cs_2_TiCl_6_-based PSCs, the present solar structure has displayed higher *J*_SC_ and FF values. The executed ETLs and HTLs in that study may not be well matched with those used in prior conducted experimental investigations, which is considered a possible reason for the discrepancy. What is more, the difference in optical properties depends on the variation of absorbers, consequently influencing the absorption of SC energy. It is observed that ETL discloses outstanding PV performance according to JV and QE characteristics in that present study; the reason is to display better band alignment as compared to other studied ETLs. The carrier transportability and transparency ETL play a significant role in the device’s efficiency of extracting out and conveying charge. The CdS is ubiquitously used as an ETL in PSC configurations due to lower hysteresis factors and higher electron mobility in comparison to TiO_2_; the latter is responsible for higher extraction rates, so as device performance^113^. In addition, exceptional transparency shown by CdS acts as a vital element for amplifying the light absorption within the perovskite absorber layer^114^.

##  Conclusion

A stable, lead-free, eco-friendly, and fully inorganic perovskite solar cell (PSC) based on Cs_2_TiCl_6_was analyzed and simulated using SCAPS-1D, where Cs_2_TiCl_6_ served as the light-absorbing layer. Throughout the investigation, a diverse set of hole transport layers (HTLs) and electron transport layers (ETLs) were examined to identify the most efficient device configuration. Key findings are listed below:

1. Among the various candidates, the combination of CdS, Nb_2_O_5_, ZnSe, and MZO as ETL, paired with a CdTe HTL, demonstrated superior performance compared to other HTL/ETL combinations.
2. Initial optimization revealed that these four devices were suitable for further analysis regarding absorber characteristics, HTL thickness, donor and defect densities in the ETL, acceptor and defect densities in the HTL, series resistance (R_S_), shunt resistance (R_sh_), temperature effects, generation rate, recombination rate, J–V characteristics, and quantum efficiency (QE).
3. Notably, the CdS-based device achieved an efficiency of 14.3% after optimizing the absorber thickness, and an enhanced efficiency of 18% after optimizing the HTL thickness. The findings indicate that minimizing series resistance and maximizing shunt resistance are crucial strategies to achieve the highest possible device performance.
4. Temperature effects were also evaluated, and the maximum efficiency was observed at 300 K. Following final optimization, the ZnSe-based device exhibited superior J–V performance under specific voltage conditions.
5. The CdS- and MZO-based PSCs displayed distinctive J–V, and QE behavior, while the Nb_2_O_5_-based device showed the poorest QE performance across the visible spectrum.
6. Based on the results, the optimal device structure proposed is FTO/CdS/Cs_2_TiCl_6_/CdTe/Au, achieving a power conversion efficiency (PCE) of 18.15%, a short-circuit current density (_Jsc_) of 17.83 mA/cm², an open-circuit voltage (V_OC_) of 1.188 V, a fill factor (FF) of 89.51%, and a quantum efficiency (QE) of 99.7% in the visible region.

Overall, this comprehensive study offers valuable insights that will assist in the future design and development of lead-free, environmentally friendly Cs_2_TiCl_6_-based perovskite solar cells.

## Data Availability

The raw/processed data required to reproduce these findings cannot be shared at this time as the data also forms part of an ongoing study and are available from the corresponding author on reasonable request.
